# Use of Vitamin D in Children and Adults: Frequently Asked Questions

**DOI:** 10.4274/jcrpe.0012

**Published:** 2018-11-29

**Authors:** Gül Yeşiltepe Mutlu, Şükrü Hatun

**Affiliations:** 1 Koç University Hospital, Clinic of Pediatric Endocrinology and Diabetes, İstanbul, Turkey; 2 Koç University Faculty of Medicine, Department of Pediatric Endocrinology and Diabetes, İstanbul, Turkey

**Keywords:** Vitamin D, deficiency, maintenance

## Abstract

In recent years, the increase in interest and use of vitamin D has been attributed mainly to the extra-skeletal effects of vitamin D and confusion about normal reference values for serum 25-hydroxy vitamin D (25-OHD). However, The Institute of Medicine, which determines daily intake of nutrients, vitamins and minerals in the United States, emphasizes that there is no additional benefit of having a 25-OHD level above 20 ng/mL in terms of parathyroid hormone suppression, calcium absorption and “fall risk”. Taking into consideration that there has not been a significant increase in vitamin D deficiency and related conditions in Turkey over the past five years, it is not hard to suppose that this increased interest is due to doctors, using mass media platforms, who have made claims that vitamin D is a “panacea”. This paper aims to answer some frequently asked questions such as the threshold values recommended for the evaluation of vitamin D status, the clinical indications for measuring 25-OHD and suggestions on the use of lifelong vitamin D starting from pregnancy.

## Introduction

Over the last 10 years an increasing interest in vitamin D deficiency and its effect, not only on extra-skeletal tissues but also on general human health, has been observed not only in Turkey but all over the world.

Recently published research, based on data of 711,718 children in the UK, showed that the frequency of diagnosis of vitamin D deficiency increased from 3.14/100,000 in 2000 to 261/100,000 in 2014 and a 15-fold increase was reported after adjustment for population increase (1). However, reliable institutions and researchers have expressed the opinion that we are not confronting a vitamin D deficiency pandemic but that this rise is related to the change in diagnostic behaviour of physicians and other health care professionals, as well as to an increase in the demand for vitamin D examination during routine visits (1,2).

Severe vitamin D deficiency may result in hypocalcemic seizures and hypocalcemic cardiomyopathy in infancy. Therefore, we suggest that 25-hydoxy-vitamin D (25-OHD) levels in infant and mother should be a routine part of evaluation of infantile hypocalcemia. It should also be noted that the regulation and action of parathyroid hormone (PTH) may be disturbed by vitamin D deficiency, particularly in infancy. Elevated PTH levels associated with hypocalcemia and normal or high phosphate indicate an element of end-organ resistance to PTH, mimicking pseudo-hypoparathyroidism (3). Studies performed in the last decade detected severe vitamin D deficiency (<10 ng⁄mL) in 46-80% of pregnant women and nursing mothers in different regions of Turkey (4,5,6,7,8). Low socioeconomic status, covered clothing style, low educational level and spending less time outdoors because of cultural and lifestyle factors are associated with maternal and perinatal vitamin D deficiency (4,5,6,7,8). There is no doubt that the only way of preventing vitamin D deficiency and its complications is vitamin D supplementation.

In addition nationwide data demonstrating increase in frequency of vitamin D deficiency over time in Turkey is not available. However, the serum 25-OHD levels of 110,774 individuals, obtained between January 2011 to December 2016 and assessed in a single laboratory serving the whole country using the liquid chromatography-tandem mass spectrometry method revealed no significant difference over time (9). Surprisingly, data obtained from Intercontinental Marketing Services Health ‘IMS Health’ showed that in 2012, 2,280,626 boxes of vitamin D (each box contains 300,000 units of vitamin D) were sold in Turkey, which rose to 8,376,319 in the first eight months of 2016. According to the same data, in 2015, only 925,734 of 8,754,753 boxes of vitamin D (less than one in ten) were prescribed.

Taking into consideration that there has been no observable increase in vitamin D deficiency-related conditions in Turkey during recent years, it is possible to assume that this increase is due to declarations of doctors, appearing via the mass media, who have made claims that vitamin D is a “panacea”. The Turkish National Pediatric Endocrinology and Diabetes Society was concerned enough that it released a statement about the harm that these physicians might cause and has drawn attention to false information on vitamin D (10). This paper aims to answer some frequently asked questions, such as the threshold values recommended for the evaluation of vitamin D status and suggestions on the use of lifelong vitamin D starting from pregnancy.

## What is the reason for the increasing interest in vitamin D in recent years? What are the normal threshold values for vitamin D?

In recent years, the increase in interest and use of vitamin D has been attributed mainly to the extra-skeletal effects of vitamin D and confusion about normal reference values for serum 25-OHD and in particular The American Endocrine Society’s recommendation proposing at least 30 ng/mL for the lower limit of normal range for serum 25-OHD level (11).

The Institute of Medicine (IOM), which determines daily intake of nutrients, vitamins and minerals in the United States, emphasized that there is no additional benefit of having a 25-OHD level above 20 ng/mL in terms of PTH suppression, calcium absorption and “fall risk”. In several reports it was stated that skeletal effects of vitamin D plateau when the 25-OHD level is between 12-16 ng/mL and 25-OHD levels below 20 ng/mL should not be accepted as ‘deficiency’ in all cases. The IOM remarks ‘it is false to specify 25-OHD >30 ng/mL as the “desired” threshold and there is no need to supplement high doses of vitamin D for obese individuals’ (12,13). Recently, members of the IOM D Vitamin Committee addressed supplementing vitamin D and recommend 400 units per day in the first year of life, 600 units in the first 70 years of life, and 800 units of vitamin D after the age of 70 years. They noted that it is possible to achieve serum vitamin D levels of 16-20 ng/mL in 97.5% of the general population.

In this report the authors highlighted the misinterpretation of a value of 20 ng/mL 25-OHD as a threshold value for bone health given that 97.5% of the general population actually have a 25-OHD level equal to or below 20 ng/mL (2). This misinterpretation may lead a shifting the entire population to a higher intake and could cause the upper intake limit (4000 units of vitamin D per day) will become normal practice, which would also bring risks.

It should not be forgotten that inadequate dietary calcium intake is as important as vitamin D to the development of rickets/osteomalacia. The IOM’s recommendation for daily calcium intake is 700-1300 mg for children, 1000-1200 mg for adults (12).

## How important are extra-skeletal effects of vitamin D? Is there a need to define a different threshold of 25-OHD level for these effects?

In addition to the intestine, which is the main site of active vitamin D (calcitriol) effect, several tissues such as breast, bone marrow, nerve cells and the immune system have vitamin D receptors and it has been suggested that calcitriol plays a role in the functions of 230 different genes (14). Recently, attention has focused on the extra-skeletal effects of calcitriol and numerous studies establishing a relationship between calcitriol and many diseases (especially cancer) have appeared widely in medical journals (15). The vast majority of this research is made up of correlational studies and fails to meet the cause and effect relation criteria.

Some researchers in The United States claim milder deficiency of vitamin D causes a predisposition to diseases of extra-skeletal tissues (15). Those publications have caused concern in the community by highlighting the supposed risks associated with 25-OHD levels below 30 ng/mL and they have encouraged healthy people to check vitamin D levels and to take high doses of vitamin D (15).

However, data from vitamin D receptor knock-out animal studies indicated the effects of calcitriol on extra-skeletal tissues were not significant, yet entirely confirmed its effects on calcium absorption and indirect effects through calcium supply on bone texture (16). Besides, a study carried out in human with hereditary vitamin D resistant rickets showed that calcium absorption was highly dependent on vitamin D from infancy until the end of puberty, however hereditary 1.25-dihydroxyvitamin-D-resistant rickets patients have normal plasma renin activity, without any indications of hypertension or gross heart abnormities, such as reduced contractility or hypertrophy, at least until the age of 37 years (17).

The IOM remarked that the outcomes of studies that relate the level of vitamin D to non-skeletal pathologies such as cancer, cardiovascular disease, diabetes and auto-immune diseases are not consistent with each other and do not require establishing a different 25-OHD threshold or a higher intake of vitamin D to prevent these diseases (12,13).

From a clinical point of view, an increase in problems expected from extra-skeletal effects of vitamin D deficiency in countries/regions/groups where vitamin D deficiency is frequent has not been reported. For instance, there are no reports of a high frequency of type 1 diabetes among children who had rickets. The relationship between vitamin D deficiency and the occurrence of type 1 diabetes has almost been a “cliché” and this information is often regarded as correct because it has been repeated for many years. Recent research in Finland has shown that there is no association between type 1 diabetes antibody positivity, the development of clinical type 1 diabetes and serum 25-OHD levels (18).

In conclusion, studies on the effects of vitamin D and extra-skeletal effects do not provide coherent data and the recommendation for 25-OHD level to be at least 30 ng/mL to obtain protective effects is unproven.

## Does total 25-OHD show the whole truth? Is it necessary to supplement a high dose of vitamin D in obese children and adolescents?

Approximately 80% of total 25-OHD is transported by vitamin D binding protein (VDBP), which has a half-life of 1-2 days. It is known that VDBP is a negative acute phase reactant and in cases such as sepsis, synthesis in the liver decreases and therefore total 25-OHD is found to be low (19). A study from the USA showed that total 25-OHD levels in African-American women were associated with low VDBP, so that African-American women and Caucasian women had similar “bioavailable” D vitamin levels (20). In another study, despite low levels of total 25-OHD in obese children, the bioavailable D vitamin level was shown to be normal and there was a negative correlation between insulin resistance and VDBP (21). Similarly, research from Turkey revealed no relationship between insulin resistance parameters and vitamin D levels in obese children (22).

It is well known that 25-OHD levels are generally low in obese people, however these recover with weight loss and vitamin D requirements are not different from non-obese people (13).

On the basis of this evidence, there is no need to routinely monitor serum vitamin D level in obese subjects and it is not necessary to prescribe vitamin D at doses higher than 400 IU per day to enhance the low levels of 25-OHD levels found.

## Is routine vitamin D testing and/or intake of vitamin D ampoules necessary for healthy people?

Overall, when vitamin D deficiency is severe (serum 25-OHD level ≤12 ng/mL) bone metabolism deteriorates and diseases such as rickets in children and osteomalacia in adults result (23).

In Turkey, a nationwide ‘vitamin D prophylaxis augmentation programme’ was initiated in 2005 using a simple but effective method which included free distribution of vitamin D drops to all new-borns and infants (0-12 months) visiting primary healthcare stations throughout the country. This programme has reduced the number of clinical rickets cases and the incidence of severe vitamin D deficiencies dramatically in Turkey (24). There is absolutely no need to test vitamin D levels in routine follow-up and to prescribe high doses of vitamin D because of low vitamin D levels in infancy and childhood. Indeed, the Global Consensus clearly states that testing is not indicated in asymptomatic individuals (23). Instead, all infants from birth, all pregnant women and all ethnic/cultural risk groups require supplementation. Nevertheless, the frequency of serum 25-OHD testing has increased approximately 2.60 times in the 0-18 years old age group and 32% in the over 18s between 2011-2016 (4).

Adults with osteomalacia might suffer from widespread bone pain and muscle weakness, particularly in the vertebrae, when vitamin D concentrations drop to 12 ng/mL or less. Therefore, neither routinely testing vitamin D in healthy, aymptomatic subjects over 40 years of age, nor prescription/intake of high dose vitamin D for serum concentrations of vitamin D below 20 ng/mL is required. This is because serum concentrations of vitamin D are only a biochemical parameter and do not give the whole picture. It is necessary to test serum alkaline phosphatase (ALP) and PTH concentrations and make an assessment based on these findings and other clinical and radiological findings to diagnose the disease.

## Has the definition of vitamin D deficiency changed? Who should have been treated with high dose vitamin D? Is testing only serum 25-OHD enough to make a decision?

Thresholds used for vitamin D deficiency differ in children and adults, but many physicians tend to interpret values below 20 ng/mL as deficiency regardless of patient age.

 In children, the laboratory 25-OHD threshold for vitamin D deficiency is 12 ng/mL and 12-20 ng/mL for insufficiency. Serum 25-OHD values above 20 ng/mL are accepted as vitamin D sufficiency (Table 1) (23). However, in The Endocrine Society’s 2011 guidelines, a 25-OHD level below 20 ng/mL is defined as deficiency and a level between 20-30 ng/mL is defined as insufficiency (11). This older recommendation has led physicians to administer high dose vitamin D treatment. It is also outdated in relation to the musculoskeletal effects of vitamin D. A similar recommendation exists in the guidelines of The Association of Adult Endocrinology and Metabolism in Turkey and administering “vitamin D stoss therapy” is recommended if the serum 25-OHD level is <20 ng/mL (25). In contrast to The Endocrine Society’s 2011 and The Association of Adult Endocrinology and Metabolism in Turkey’s recommendations the IOM consider that an intake of 400 IU/day of vitamin D is adequate in order to ensure a serum vitamin D level between 16 and 20 ng/mL (12). In addition, in a CDC report analysing vitamin D status in the US, the threshold serum 25-OHD level was taken as 12 ng/mL as a definition of vitamin D deficiency and it was reported that levels above 50 ng/mL are ‘possibly harmful’ (26). Furthermore, it is essential to confirm an elevation of serum ALP and/or PTH before administration of vitamin D at the treatment dose. Treatment dose is determined as a peroral, single dose of 50,000 IU vitamin D for children aged 3-12 months, 150,000 IU for children aged 12 months to 12 years and 300,000 IU for those aged >12 years in The European Society for Pediatric Endocrinology’s Global Consensus Recommendations on Prevention and Management of Nutritional Rickets (23).

As mentioned above, vitamin D stoss therapy (a single high dose vitamin D) or 2,000-6,000 IU/day vitamin D administration, based only on serum vitamin D level, is not advisable.

Seasonal variations of 25-OHD level should also be taken into consideration when vitamin D status is being assessed. A seasonal decline in serum 25-OHD levels has been well documented from summer to winter in two large scaled studies from different regions of Turkey (27,28).

## What is the lifelong daily maintenance dose of vitamin D? Is vitamin D supplementation needed during pregnancy?

Lifelong daily vitamin D requirements are regularly updated by the IOM in the United States. These updates include the amount that meets at least 97.5% of the healthy target population [Recommended Dietary Allowance (RDA)] and the maximum amount that can be taken per day without any risk [Upper Intake Level (UL)]. The last update from 2011 specified the RDA for vitamin D was 400 IU/day in the first year of life (UL was 1000 IU/day for infants >6 month-old, 1500 IU/day for infants 6 months-1 year) and 600 IU/day for individuals between one and 71 years (UL was 2500 IU/day for children between 1 and 3 years, 3000 IU/day for children between 4 and 8 years and 4000 IU/day for individuals >8 years) and 800 IU for individuals >71 years old (7). A recent global consensus report has recommended that 400 IU of vitamin D be given orally to all infants until one year of age (23). The IOM’s recommended dose for supplementing vitamin D in pregnancy is 600 IU/day (UL: 4000 IU). D-vitamin supplementation during pregnancy is primarily required for the prevention of late hypocalcemia in the new-born period. In countries where maternal vitamin D deficiency is common, such as Turkey, a dose of 1200 IU/day or more is recommended (29).

400 IU/day vitamin D for new-borns (from the first day of life) and 1200 IU/day vitamin D for women from the third month of pregnancy and during lactation is recommended through the national program for the prevention of vitamin D deficiency in Turkey (30,31).

It is considered that supplementation of vitamin D in the form of oral drops until at least the first year of life, and preferably up to the age of three years, is sufficient.

Sunlight exposure, 30 minutes per week with only diaper and at least two hours per week when they are fully clothed, is also sufficient for babies after six months of age to have a vitamin D level at 11 ng/mL, however the duration of sunlight exposure that is necessary for infants and children to maintain vitamin D levels at 50 nmol/L (20 ng/mL) in children remains to be determined. In the meantime, it is necessary to keep in mind that sunscreens and sunlight exposure through glass reduce the synthesis of vitamin D by more than 90% (32,33).

## What are the main incorrect attitudes regarding use of vitamin D in children?

In Turkey, vitamin D deficiency rickets was a common problem in the first two years of life in the past. Affected infants had signs such as delayed walking and teething so that some families, pharmacists and sometimes physicians had a tendency to make toddlers drink vitamin D ampoules with the idea of “earlier walking” and “earlier teething” due to this association.

However, supplementing a baby with higher doses of vitamin D than required has no effect on early walking and teething. Beyond that, it may result in permanent damage by causing “vitamin D intoxication” and renal calcifications.

Another misconception is the administration of high dose D vitamin to children with bowed legs without thorough examination. Bowed legs may be seen in vitamin D deficiency rickets but it is not the only etiology of leg bowing which include physiologic bowing and genetic skeletal disorders. Thus, children with bowed legs should not be randomly given high dose vitamin D and any such cases should definitely be examined by a pediatric endocrinologist.

Finally, some physicians in Turkey discontinue supplementing vitamin D in the first few months of life because of “small” fontanelles. This is another misconception because closure of the fontanelles is delayed in the case of vitamin D deficiency and normal or even high doses of vitamin D are not associated with early closure or smallness of the fontanelles.

## Figures and Tables

**Table 1:**
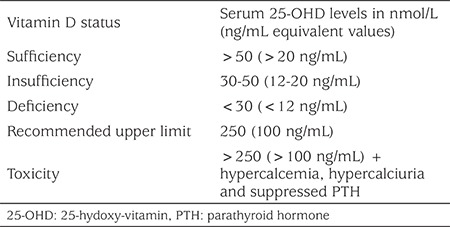
Classification of vitamin D status, based on serum 25-OHD levels (22)
